# Retraction: Kinetics of Antibody Response in BALB/c and C57BL/6 Mice Bitten by *Phlebotomus papatasi*

**DOI:** 10.1371/journal.pntd.0011856

**Published:** 2023-12-22

**Authors:** The PLOS Neglected Tropical Diseases Editors

Following the publication of this article [1], concerns were raised regarding results presented in Figs 3 and 4. Specifically,

- In Fig 3, the BALB/c Negative and C57BI/6 Negative results appear similar.
- In Fig 4A, the third and fifth bitten mice panels of the Salivary glands result appear similar.

The corresponding author stated that the results presented in Fig 3 were pooled from two experiments and that representative strips for each condition were presented in the article. Regarding the concerns with Fig 4, the corresponding author stated that differences between the indicated lanes can be observed with higher contrast and magnification.

Underlying data for Figs 3 and 4 were provided by the corresponding author. PLOS assessed the comments and data provided and remains concerned that the panels listed above are more similar than would be expected from independent data. In addition, during editorial assessment of the underlying data, mismatches were detected between these data and their corresponding figure panels, which raised further concerns about the reliability and integrity of the published results.

In light of these concerns the *PLOS Neglected Tropical Diseases* Editors retract this article.

JGV agreed with the retraction. MV, IR, JH, LZ, JD, and PV did not agree with the retraction and stand by the article’s findings. LP either did not respond directly or could not be reached.
